# Crystal structure and Hirshfeld surface analysis of (*E*)-5-phenyl-3-[(pyridin-4-yl­methyl­idene)amino]­thia­zolidin-2-iminium bromide monohydrate

**DOI:** 10.1107/S2056989018011155

**Published:** 2018-08-21

**Authors:** Mehmet Akkurt, Abel M. Maharramov, Gulnara Sh. Duruskari, Flavien A. A. Toze, Ali N. Khalilov

**Affiliations:** a Department of Physics, Faculty of Sciences, Erciyes University, 38039 Kayseri, Turkey; b Organic Chemistry Department, Baku State University, Z. Xalilov str. 23, Az, 1148 Baku, Azerbaijan; c Department of Chemistry, Faculty of Sciences, University of Douala, PO Box 24157, Douala, Republic of Cameroon

**Keywords:** crystal structure, charge-assisted hydrogen bonding, pyridine ring, thia­zolidine ring, Hirshfeld surface analysis

## Abstract

In the cation of the title salt, C_15_H_15_N_4_S^+^·Br^−^·H_2_O, the central thia­zolidine ring adopts an envelope conformation with puckering parameters *Q*(2) = 0.279 (4) Å and φ(2) = 222.5 (9)°. The mean plane of the thia­zolidine ring makes dihedral angles of 12.4 (2) and 66.8 (3)° with the pyridine and phenyl rings, respectively. The pyridine ring in the title mol­ecule is essentially planar (r.m.s deviation = 0.005 Å). In the crystal, the cations, anions and water mol­ecules are linked into a three-dimensional network, which forms cross layers parallel to the (120) and (20) planes *via* O—H⋯Br, N—H⋯Br and N—H⋯N hydrogen bonds. C—H⋯π inter­actions also help in the stabilization of the mol­ecular packing. Hirshfeld surface analysis and 2D (two-dimensional) fingerprint plots indicate that the most important contributions to the crystal packing are from H⋯H (35.5%), C⋯H/H⋯C (23.9%), Br⋯H/H⋯Br (16.4%), N⋯H/H⋯N (10.6%) and S⋯H/H⋯S (7.9%) inter­actions.

## Abstract 2

In the crystal structure of the title salt, the cations, anions and water mol­ecules are linked into a three-dimensional network, which forms cross layers parallel to the (120) and (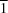
20) planes *via* O—H⋯Br, N—H⋯Br and N—H⋯N hydrogen bonds. C—H⋯π inter­actions also help in the stabilization of the mol­ecular packing.

## Chemical context   

Schiff bases and related hydrazone compounds play an important role in coordination and medicinal chemistry due to their high coordination ability (Mahmoudi *et al.*, 2017*a* ▸,*b* ▸,*c* ▸; Mitoraj *et al.*, 2018 ▸; Shixaliyev *et al.*, 2013*a* ▸), application of those metal complexes in catalysis (Jlassi *et al.*, 2014 ▸; Gurbanov *et al.*, 2018 ▸; Mahmudov *et al.*, 2014 ▸; Shixaliyev *et al.*, 2013*b* ▸, 2014 ▸), biological properties (Abedi *et al.*, 2014 ▸), *etc*. Inter- and intra­molecular weak inter­actions may also effect their properties (Mahmudov *et al.*, 2016 ▸, 2017*a* ▸,*b* ▸). Herein we found strong O—H⋯Br^−^ and N^+^—H⋯Br^−^ types of charge-assisted hydrogen bonds in (*E*)-5-phenyl-3-[(pyridin-4-yl­methylid­ene)amino]­thia­zolidin-2-iminium bromide monohy­drate.
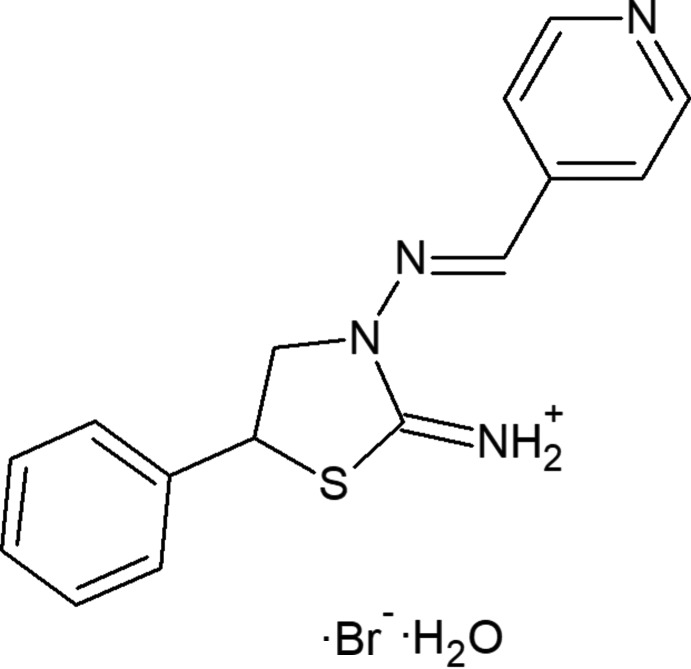


## Structural commentary   

**Figure 1:**
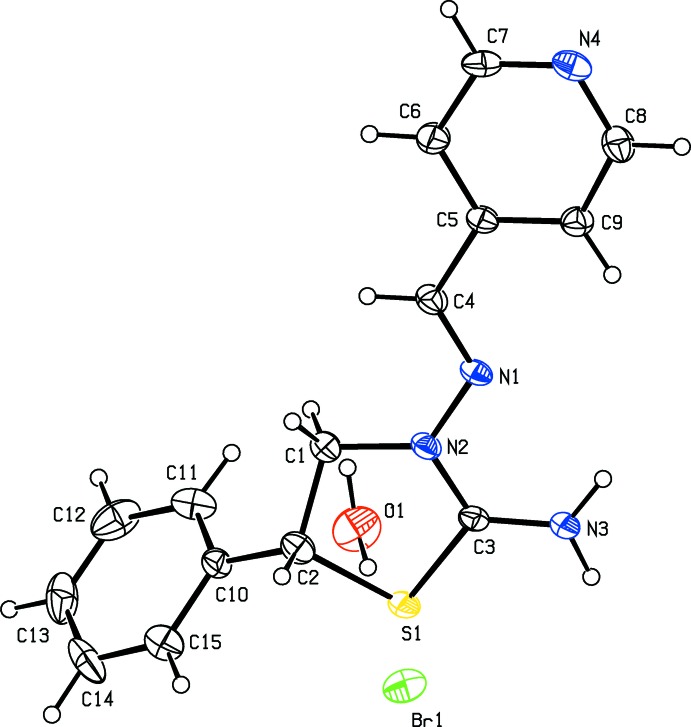
The mol­ecular structure of the title salt. Displacement ellipsoids are drawn at the 30% probability level. H atoms are shown as spheres of arbitrary radius.

The thia­zolidine ring (atoms S1/C1–C4) in the cation of the title salt (Fig. 1 ▸) adopts an envelope conformation with the puckering parameters (Cremer & Pople, 1975 ▸) *Q*(2) = 0.279 (4) Å and φ(2) = 222.5 (9)°. The mean plane of the thia­zolidine ring makes dihedral angles of 12.4 (2) and 66.8 (3)° with the pyridine (N4/C5–C9) and phenyl (C10–C15) rings, respectively. The pyridine ring of the title mol­ecule is essentially planar (r.m.s deviation = 0.005 Å). The N2—N1—C4—C5 bridge that links the thia­zolidine and 2,3-di­chloro­benzene rings has a torsion angle of 178.3 (4)°.

## Supra­molecular features and Hirshfeld surface analysis   

**Figure 2:**
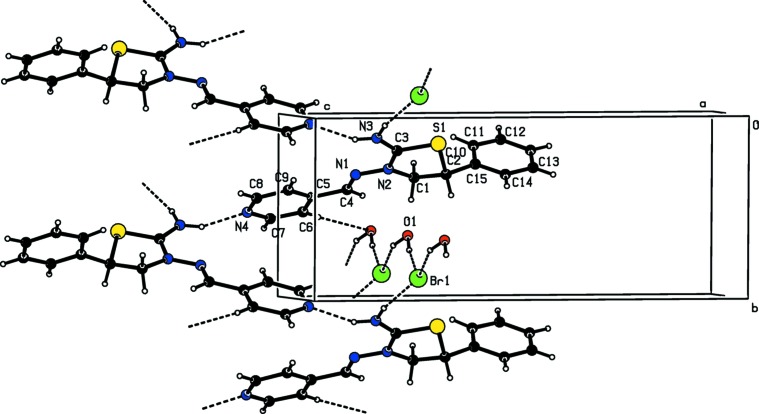
A view of the inter­molecular hydrogen bonds of the title compound along the *a* axis.

As shown in Figs. 2 ▸ and 3 ▸, in the crystal, the cations, anions and water mol­ecules are linked into a three-dimensional network, which forms cross layers parallel to the (120) and (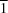
20) planes *via* O—H⋯Br, N—H⋯Br and N—H⋯N hydrogen bonds (Table 1 ▸). Furthermore, C—H⋯π inter­actions also help in the stabilization of the mol­ecular packing (Table 1 ▸).

**Figure 4:**
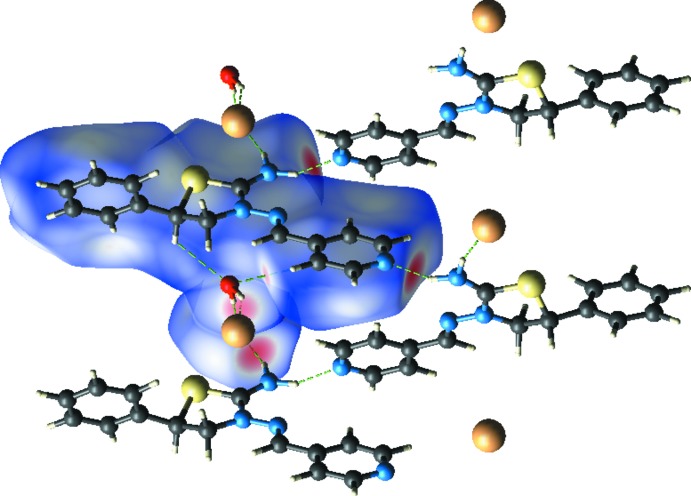
Hirshfeld surface of the title compound mapped over *d*_norm_.

Hirshfeld surface analysis was used to investigate the presence of hydrogen bonds and inter­molecular inter­actions in the crystal structure. The Hirshfeld surface analysis (Spackman & Jayatilaka, 2009 ▸) of the title salt was generated by *CrystalExplorer3.1* (Wolff *et al.*, 2012 ▸), and comprised *d*_norm_ surface plots and 2D (two-dimensional) fingerprint plots (Spackman & McKinnon, 2002 ▸). The plots of the Hirshfeld surface mapped over *d*_norm_ using a standard surface resolution with a fixed colour scale of −0.5782 (red) to 1.2417 a.u. (blue) is shown in Fig. 4 ▸. This plot was generated to qu­antify and visualize the inter­molecular inter­actions and to explain the observed crystal packing. The dark-red spots on the *d*_norm_ surface arise as a result of short inter­atomic contacts, while the other weaker inter­molecular inter­actions appear as light-red spots.

**Figure 5:**
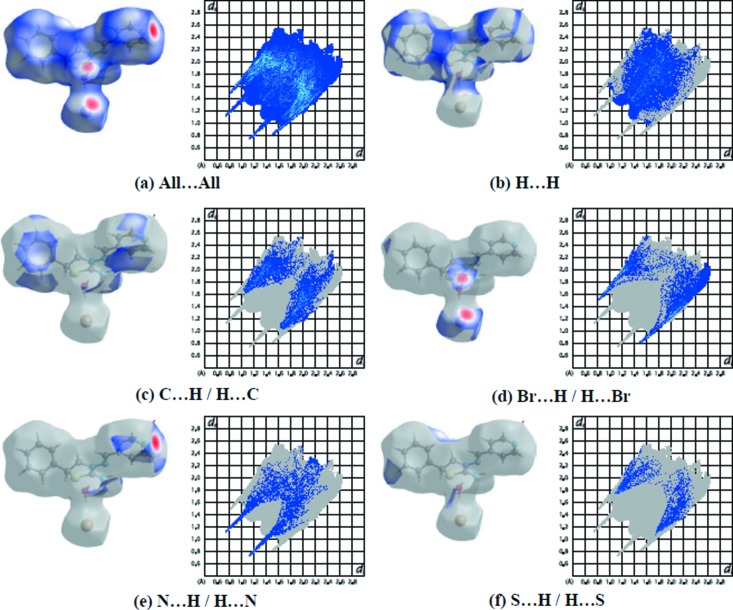
The two-dimensional fingerprint plots of the title compound, showing (*a*) all inter­actions, and delineated into (*b*) H⋯H, (*c*) C⋯H/H⋯C, (*d*) Br⋯H/H⋯Br, (*e*) N⋯H/H⋯N and (*f*) S⋯H/H⋯S inter­actions [*d*_e_ and *d*_i_ represent the distances from a point on the Hirshfeld surface to the nearest atoms outside (external) and inside (inter­nal) the surface, respectively].

Fig. 5 ▸(*a*) shows the 2D fingerprint plot of the sum of the contacts contributing to the Hirshfeld surface represented in normal mode. These represent both the overall two-dimensional fingerprint plots and those that represent H⋯H, C⋯H/H⋯C, Br⋯H/H⋯Br, N⋯H/H⋯N and S⋯H/H⋯S contacts, respectively (Figs. 5 ▸ *b–f*). The most significant inter­molecular inter­actions are the H⋯H inter­actions (35.5%) (Fig. 5 ▸ *b*). The reciprocal C⋯H/H⋯C inter­actions appear as two symmetrical broad wings with *d*_e_ + *d*_i_ ≃ 2.7 Å and contribute 23.9% to the Hirshfeld surface (Fig. 5 ▸ *c*). The reciprocal Br⋯H/H⋯Br, N⋯H/H⋯N and S⋯H/H⋯S inter­actions with 16.4, 10.6 and 7.9% contributions are present as sharp symmetrical spikes at diagonal axes *d*_e_ + *d*_i_ ≃ 2.3, 2.9 and 2.8 Å, respectively (Figs. 5 ▸ *d*–*f*). Furthermore, there are O⋯H/H⋯O (2.8%), Br⋯C/C⋯Br (1.1%), Br⋯N/N⋯Br (1.0%), Br⋯S/S⋯Br (0.6%), N⋯C/C⋯N (0.3%) and N⋯N (0.1%) contacts (Table 2 ▸).

## Database survey   

In a recent article of ours, which on the crystal structure of (*E*)-3-[(2,3-di­chloro­benzyl­idene)amino]-5-phenyl­thia­zolidin-2-iminium bromide (Akkurt *et al.*, 2018 ▸), the 3-N atom of the cation carries an N substituent, as found in the title compound. In the crystal, C—H⋯Br and N—H⋯Br hydrogen bonds link the components into a three-dimensional network with the cations and anions stacked along the *b*-axis direction. Weak C—H⋯π inter­actions and inversion-related Cl⋯Cl halogen bonds and C—Cl⋯π(ring) contacts also contribute to the mol­ecular packing.

In addition, a search of the Cambridge Structural Database (CSD Version 5.39, November 2017 + 3 updates; Groom *et al.*, 2016 ▸) yielded six hits for 2-thia­zolidiniminium compounds, with four of them reporting essentially the same cation [CSD refcodes WILBIC (Marthi *et al.*, 1994 ▸), WILBOI (Marthi *et al.*, 1994 ▸), WILBOI01 (Marthi *et al.*, 1994 ▸), YITCEJ (Martem’yanova *et al.*, 1993*a* ▸), YITCAF (Martem’yanova *et al.*, 1993*b* ▸) and YOPLUK (Marthi *et al.*, 1995 ▸)]. In all cases, the 3-N atom carries a C substituent not N as found in the title compound. The first three crystal structures were determined for racemic (WILBIC; Marthi *et al.*, 1994 ▸) and two optically active samples (WILBOI and WILBOI01; Marthi *et al.*, 1994 ▸) of 3-(2-chloro-2-phenyl­eth­yl)-2-thia­zolidiniminium *p*-tolu­ene­sulfonate. In all three structures, the most disordered fragment of these mol­ecules is the asymmetric C atom and the Cl atom attached to it. The disorder of the cation in the racemate corresponds to the presence of both enanti­omers at each site in the ratio 0.821 (3):0.179 (3). The system of hydrogen bonds connecting two cations and two anions into 12-membered rings is identical in the racemic and in the optically active crystals. YITCEJ (Martem’yanova *et al.*, 1993*a* ▸) is a product of the inter­action of 2-amino-5-methyl­thia­zoline with methyl iodide, with alkyl­ation at the endocylic N atom, while YITCAF (Martem’yanova *et al.*, 1993*b* ▸) is a product of the reaction of 3-nitro-5-meth­oxy-, 3-nitro-5-chloro- and 3-bromo-5-nitro­salicyl­aldehyde with the heterocyclic base to form the salt-like complexes.

## Synthesis and crystallization   

To the solution of 1 mmol of 3-amino-5-phenyl­thia­zolidin-2-iminium bromide in 20 ml ethanol was added 1 mmol of isonicotinaldehyde and the solution was refluxed for 2 h. The reaction mixture was then cooled. Reaction products were precipitated from the reaction mixture as colourless single crystals, collected by filtration and washed with cold acetone.

Yield: 57%; m.p.: 496 K. Analysis calculated for C_15_H_15_BrN_4_S: C 49.59, H 4.16, N 15.42%; found: C 49.52, H 4.11, N 15.35%. ^1^H NMR (300 MHz, DMSO-*d*_6_) : δ 4.57 (*q*, 1H, CH_2_, ^3^ *J*_H–H_ = 6.6 Hz), 4.89 (*t*, 1H, CH_2_, ^3^ *J*_H–H_ = 8.1 Hz), 5.62 (*t*, 1H, CH-Ar, ^3^ *J*_H–H_ = 7.5 Hz), 7.37–7.57 (*m*, 5H, 5 Ar-H), 8.015–7.998 (*d*, 2H, 2CH_arom_, ^3^ *J*_H–H_ = 5.1 Hz), 8.46 (*s*, 1H, CH=), 8.728–8.711 (*d*, 2H, 2CH_arom_, ^3^ *J*_H–H_ = 5.1 Hz), 10.52 (*s*, 2H, NH_2_=). ^13^C NMR (75MHz, DMSO-*d*_6_): δ 45.54, 56.00, 122.17, 127.86, 128.98, 129.16, 137.43, 140.16, 148.88, 150.31, 168.98. MS (ESI), *m*/*z*: 283.36 [C_15_H_15_N_4_S]^+^ and 79.88 Br^−^.

## Refinement details   

Crystal data, data collection and structure refinement details are summarized in Table 3 ▸. All H atoms were positioned geometrically and refined using a riding model, with O—H = 0.95 Å, N—H = 0.90 Å and C—H = 0.93–0.98 Å, and with *U*_iso_(H) = 1.2*U*_eq_(C,N) or 1.5*U*_eq_(O) for the H atoms of the water mol­ecule.

## Supplementary Material

Crystal structure: contains datablock(s) I, global. DOI: 10.1107/S2056989018011155/xu5936sup1.cif

Structure factors: contains datablock(s) I. DOI: 10.1107/S2056989018011155/xu5936Isup2.hkl

CCDC reference: 1837127

Additional supporting information:  crystallographic information; 3D view; checkCIF report

## Figures and Tables

**Figure 3:**
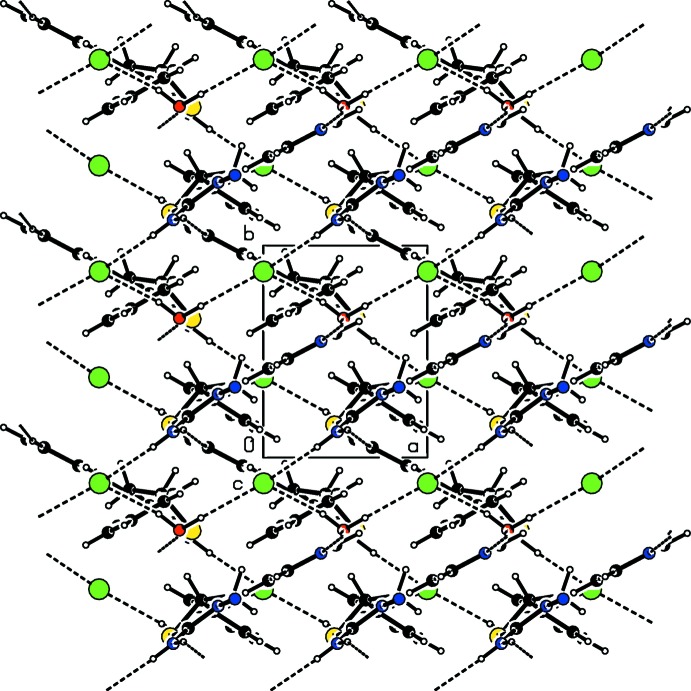
A view of the packing and inter­molecular hydrogen bonding of the title compound along the *c* axis.

**Table 1:** Hydrogen-bond geometry (Å, °) *Cg*2 and *Cg*3 are the centroids of the N4/C5–C9 pyridine and C10–C15 phenyl ring, respectively.

*D*—H⋯*A*	*D*—H	H⋯*A*	*D*⋯*A*	*D*—H⋯*A*
O1—H1*C*⋯Br1	0.95	2.39	3.333 (4)	169
O1—H1*D*⋯Br1^i^	0.95	2.56	3.427 (5)	152
N3—H3*A*⋯Br1^ii^	0.90	2.45	3.333 (4)	167
N3—H3*B*⋯N4^iii^	0.90	1.99	2.840 (6)	158
C9—H9*A*⋯*Cg*2^iii^	0.93	2.96	3.650 (5)	132
C12—H12*A*⋯*Cg*3^iv^	0.93	2.82	3.565 (8)	137
C15—H15*A*⋯*Cg*3^v^	0.93	2.80	3.548 (6)	135

Symmetry codes: (i) 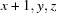; (ii) 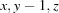; (iii) 
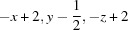
; (iv) 
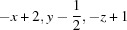
; (v) 
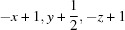
.

**Table 2:** Percentage contributions of inter­atomic contacts to the Hirshfeld surface for the title salt

Contact	Percentage contribution
H⋯H	35.5
C⋯H/H⋯C	23.9
Br⋯H/H⋯Br	16.4
N⋯H/H⋯N	10.6
S⋯H/H⋯S	7.9
Br⋯C/C⋯Br	1.1
Br⋯N/N⋯Br	1.0
Br⋯S/S⋯Br	0.6
C⋯N/N⋯C	0.3
N⋯N/N⋯N	0.1

**Table 3:** Experimental details

Crystal data	
Chemical formula	C_15_H_15_N_4_S^+^·Br^−^·H_2_O
*M*_r_	381.30
Crystal system, space group	Monoclinic, *P*2_1_
Temperature (K)	296
*a*, *b*, *c* (Å)	5.8515 (8), 7.5304 (10), 18.859 (3)
β (°)	93.979 (5)
*V* (Å^3^)	829.0 (2)
*Z*	2
Radiation type	Mo *K*α
μ (mm^−1^)	2.61
Crystal size (mm)	0.19 × 0.15 × 0.14

Data collection	
Diffractometer	Bruker APEXII CCD
Absorption correction	Multi-scan (*SADABS*; Bruker, 2007 ▸)
*T*_min_, *T*_max_	0.623, 0.698
No. of measured, independent and observed [*I* > 2σ(*I*)] reflections	12061, 3373, 3107
*R*_int_	0.076
(sin θ/λ)_max_ (Å^−1^)	0.626

Refinement	
*R*[*F* ^2^ > 2σ(*F* ^2^)], *wR*(*F* ^2^), *S*	0.037, 0.087, 1.08
No. of reflections	3373
No. of parameters	199
No. of restraints	1
H-atom treatment	H-atom parameters constrained
Δρ_max_, Δρ_min_ (e Å^−3^)	0.49, −0.47
Absolute structure	Flack *x* determined using 1291 quotients [(*I* ^+^) − (*I* ^−^)]/[(*I* ^+^) + (*I* ^−^)] (Parsons *et al.*, 2013 ▸)
Absolute structure parameter	0.004 (8)

Computer programs: *APEX2* and *SAINT* (Bruker, 2007 ▸), *SHELXS97* (Sheldrick, 2008 ▸), *SHELXL2014* (Sheldrick, 2015 ▸), *ORTEP-3 for Windows* (Farrugia, 2012 ▸) and *PLATON* (Spek, 2009 ▸).

## supplementary crystallographic information

### Crystal data

**Table:**

C_15_H_15_N_4_S^+^·Br^−^·H_2_O	*F*(000) = 388
*M**_r_* = 381.30	*D*_x_ = 1.527 Mg m^−^^3^
Monoclinic, *P*2_1_	Mo *K*α radiation, λ = 0.71073 Å
*a* = 5.8515 (8) Å	Cell parameters from 6867 reflections
*b* = 7.5304 (10) Å	θ = 2.9–26.4°
*c* = 18.859 (3) Å	µ = 2.61 mm^−^^1^
β = 93.979 (5)°	*T* = 296 K
*V* = 829.0 (2) Å^3^	Block, colorless
*Z* = 2	0.19 × 0.15 × 0.14 mm

### Data collection

**Table:**

Bruker APEXII CCD diffractometer	3107 reflections with *I* > 2σ(*I*)
φ and ω scans	*R*_int_ = 0.076
Absorption correction: multi-scan (SADABS; Bruker, 2007)	θ_max_ = 26.4°, θ_min_ = 2.9°
*T*_min_ = 0.623, *T*_max_ = 0.698	*h* = −7→7
12061 measured reflections	*k* = −9→9
3373 independent reflections	*l* = −21→23

### Refinement

**Table:**

Refinement on *F*^2^	Secondary atom site location: difference Fourier map
Least-squares matrix: full	Hydrogen site location: mixed
*R*[*F*^2^ > 2σ(*F*^2^)] = 0.037	H-atom parameters constrained
*wR*(*F*^2^) = 0.087	*w* = 1/[σ^2^(*F*_o_^2^) + (0.0256*P*)^2^ + 0.6286*P*] where *P* = (*F*_o_^2^ + 2*F*_c_^2^)/3
*S* = 1.08	(Δ/σ)_max_ < 0.001
3373 reflections	Δρ_max_ = 0.49 e Å^−^^3^
199 parameters	Δρ_min_ = −0.47 e Å^−^^3^
1 restraint	Absolute structure: Flack *x* determined using 1291 quotients [(I+)-(I-)]/[(I+)+(I-)] (Parsons *et al.*, 2013)
Primary atom site location: structure-invariant direct methods	Absolute structure parameter: 0.004 (8)

### Special details

**Table:**

Geometry. All esds (except the esd in the dihedral angle between two l.s. planes) are estimated using the full covariance matrix. The cell esds are taken into account individually in the estimation of esds in distances, angles and torsion angles; correlations between esds in cell parameters are only used when they are defined by crystal symmetry. An approximate (isotropic) treatment of cell esds is used for estimating esds involving l.s. planes.

### Fractional atomic coordinates and isotropic or equivalent isotropic displacement parameters (Å^2^)

**Table:**

	*x*	*y*	*z*	*U*_iso_*/*U*_eq_	
Br1	0.00107 (9)	0.87805 (8)	0.76086 (3)	0.05140 (19)	
O1	0.4881 (7)	0.6571 (7)	0.7445 (3)	0.0670 (13)	
H1D	0.619040	0.714648	0.766487	0.100*	
H1C	0.362040	0.733228	0.751357	0.100*	
S1	0.4339 (2)	0.15399 (19)	0.67953 (6)	0.0382 (3)	
N1	0.8263 (6)	0.3319 (5)	0.8366 (2)	0.0295 (9)	
N2	0.7239 (7)	0.2983 (5)	0.7699 (2)	0.0303 (8)	
N3	0.4501 (6)	0.1215 (6)	0.8202 (2)	0.0355 (9)	
H3A	0.328282	0.049581	0.812009	0.043*	
H3B	0.512382	0.129791	0.865099	0.043*	
N4	1.3503 (8)	0.5477 (6)	1.0413 (2)	0.0411 (10)	
C1	0.8150 (8)	0.3485 (7)	0.7026 (2)	0.0355 (11)	
H1A	0.942871	0.272508	0.692765	0.043*	
H1B	0.867805	0.470588	0.704580	0.043*	
C2	0.6215 (9)	0.3270 (6)	0.6453 (3)	0.0367 (11)	
H2A	0.535751	0.438691	0.640770	0.044*	
C3	0.5413 (7)	0.1933 (6)	0.7656 (2)	0.0280 (9)	
C4	1.0129 (8)	0.4175 (6)	0.8403 (3)	0.0342 (11)	
H4A	1.078821	0.452819	0.799119	0.041*	
C5	1.1255 (8)	0.4610 (6)	0.9111 (2)	0.0303 (9)	
C6	1.3363 (9)	0.5471 (7)	0.9143 (3)	0.0399 (12)	
H6A	1.405181	0.577708	0.872999	0.048*	
C7	1.4407 (9)	0.5859 (8)	0.9808 (3)	0.0415 (12)	
H7A	1.582539	0.642081	0.982961	0.050*	
C8	1.1475 (10)	0.4651 (8)	1.0373 (3)	0.0421 (12)	
H8A	1.081980	0.437142	1.079457	0.051*	
C9	1.0312 (8)	0.4194 (6)	0.9743 (3)	0.0349 (11)	
H9A	0.890877	0.361247	0.974094	0.042*	
C10	0.6939 (9)	0.2728 (7)	0.5720 (3)	0.0354 (11)	
C11	0.8870 (10)	0.1764 (8)	0.5602 (3)	0.0501 (14)	
H11A	0.977907	0.131751	0.598504	0.060*	
C12	0.9462 (10)	0.1458 (11)	0.4924 (5)	0.064 (2)	
H12A	1.081948	0.086515	0.485002	0.077*	
C13	0.8064 (13)	0.2022 (10)	0.4350 (3)	0.0626 (19)	
H13A	0.846758	0.180147	0.389002	0.075*	
C14	0.6057 (15)	0.2916 (9)	0.4462 (3)	0.0610 (19)	
H14A	0.508677	0.327949	0.407801	0.073*	
C15	0.5505 (11)	0.3266 (7)	0.5146 (3)	0.0450 (13)	
H15A	0.415794	0.386965	0.522268	0.054*	

### Atomic displacement parameters (Å^2^)

**Table:**

	*U*^11^	*U*^22^	*U*^33^	*U*^12^	*U*^13^	*U*^23^
Br1	0.0357 (2)	0.0535 (3)	0.0647 (4)	0.0010 (3)	0.0019 (2)	−0.0076 (4)
O1	0.059 (3)	0.059 (3)	0.084 (4)	0.016 (3)	0.013 (2)	−0.008 (3)
S1	0.0326 (5)	0.0560 (8)	0.0249 (5)	−0.0080 (6)	−0.0050 (4)	−0.0042 (5)
N1	0.0331 (19)	0.029 (2)	0.0250 (18)	−0.0004 (14)	−0.0079 (14)	−0.0049 (13)
N2	0.0325 (19)	0.0329 (19)	0.0245 (19)	−0.0024 (16)	−0.0055 (15)	−0.0013 (15)
N3	0.0296 (18)	0.051 (3)	0.0252 (19)	−0.0036 (19)	−0.0033 (15)	0.0017 (18)
N4	0.043 (2)	0.043 (3)	0.036 (2)	0.003 (2)	−0.0098 (18)	−0.0099 (19)
C1	0.037 (2)	0.042 (3)	0.028 (2)	−0.002 (2)	−0.0003 (17)	−0.001 (2)
C2	0.040 (2)	0.034 (3)	0.035 (3)	0.0068 (19)	−0.002 (2)	−0.0006 (18)
C3	0.0238 (19)	0.031 (2)	0.028 (2)	0.0050 (18)	−0.0030 (16)	−0.0005 (18)
C4	0.038 (2)	0.037 (3)	0.027 (2)	−0.0021 (19)	−0.0028 (18)	−0.0039 (18)
C5	0.032 (2)	0.030 (2)	0.029 (2)	−0.0017 (18)	−0.0043 (18)	−0.0047 (18)
C6	0.039 (3)	0.046 (3)	0.034 (3)	−0.009 (2)	0.000 (2)	−0.006 (2)
C7	0.031 (2)	0.047 (3)	0.045 (3)	−0.003 (2)	−0.009 (2)	−0.011 (2)
C8	0.050 (3)	0.047 (3)	0.030 (3)	0.000 (2)	0.004 (2)	−0.004 (2)
C9	0.033 (2)	0.037 (3)	0.035 (2)	−0.0021 (18)	−0.0004 (18)	−0.0032 (18)
C10	0.038 (2)	0.040 (3)	0.027 (2)	−0.007 (2)	−0.0022 (19)	0.0002 (19)
C11	0.044 (3)	0.048 (3)	0.056 (3)	0.005 (3)	−0.014 (3)	−0.007 (3)
C12	0.045 (3)	0.067 (4)	0.084 (5)	−0.005 (4)	0.018 (3)	−0.031 (4)
C13	0.093 (5)	0.061 (4)	0.036 (3)	−0.017 (4)	0.026 (3)	−0.010 (3)
C14	0.103 (6)	0.045 (3)	0.032 (3)	−0.007 (4)	−0.019 (3)	0.012 (2)
C15	0.051 (3)	0.037 (3)	0.046 (3)	0.006 (2)	−0.005 (2)	−0.003 (2)

### Geometric parameters (Å, º)

**Table:**

O1—H1D	0.9500	C5—C9	1.384 (7)
O1—H1C	0.9500	C5—C6	1.391 (7)
S1—C3	1.725 (4)	C6—C7	1.389 (7)
S1—C2	1.848 (5)	C6—H6A	0.9300
N1—C4	1.266 (6)	C7—H7A	0.9300
N1—N2	1.380 (5)	C8—C9	1.372 (7)
N2—C3	1.327 (6)	C8—H8A	0.9300
N2—C1	1.459 (6)	C9—H9A	0.9300
N3—C3	1.310 (6)	C10—C11	1.374 (8)
N3—H3A	0.9000	C10—C15	1.384 (7)
N3—H3B	0.9000	C11—C12	1.367 (10)
N4—C7	1.322 (8)	C11—H11A	0.9300
N4—C8	1.337 (8)	C12—C13	1.378 (11)
C1—C2	1.519 (6)	C12—H12A	0.9300
C1—H1A	0.9700	C13—C14	1.383 (11)
C1—H1B	0.9700	C13—H13A	0.9300
C2—C10	1.529 (7)	C14—C15	1.376 (9)
C2—H2A	0.9800	C14—H14A	0.9300
C4—C5	1.484 (6)	C15—H15A	0.9300
C4—H4A	0.9300		
			
H1D—O1—H1C	106.0	C7—C6—C5	118.0 (5)
C3—S1—C2	91.2 (2)	C7—C6—H6A	121.0
C4—N1—N2	117.6 (4)	C5—C6—H6A	121.0
C3—N2—N1	117.5 (4)	N4—C7—C6	123.8 (5)
C3—N2—C1	116.3 (4)	N4—C7—H7A	118.1
N1—N2—C1	125.7 (4)	C6—C7—H7A	118.1
C3—N3—H3A	118.3	N4—C8—C9	123.4 (5)
C3—N3—H3B	123.4	N4—C8—H8A	118.3
H3A—N3—H3B	117.9	C9—C8—H8A	118.3
C7—N4—C8	117.3 (4)	C8—C9—C5	119.0 (5)
N2—C1—C2	106.9 (4)	C8—C9—H9A	120.5
N2—C1—H1A	110.3	C5—C9—H9A	120.5
C2—C1—H1A	110.3	C11—C10—C15	119.2 (5)
N2—C1—H1B	110.3	C11—C10—C2	124.8 (5)
C2—C1—H1B	110.3	C15—C10—C2	116.0 (5)
H1A—C1—H1B	108.6	C12—C11—C10	120.4 (6)
C1—C2—C10	115.6 (4)	C12—C11—H11A	119.8
C1—C2—S1	104.9 (3)	C10—C11—H11A	119.8
C10—C2—S1	109.6 (3)	C11—C12—C13	120.5 (6)
C1—C2—H2A	108.8	C11—C12—H12A	119.8
C10—C2—H2A	108.8	C13—C12—H12A	119.8
S1—C2—H2A	108.8	C12—C13—C14	119.6 (6)
N3—C3—N2	124.7 (4)	C12—C13—H13A	120.2
N3—C3—S1	121.8 (4)	C14—C13—H13A	120.2
N2—C3—S1	113.5 (3)	C15—C14—C13	119.6 (6)
N1—C4—C5	119.3 (4)	C15—C14—H14A	120.2
N1—C4—H4A	120.3	C13—C14—H14A	120.2
C5—C4—H4A	120.3	C14—C15—C10	120.5 (6)
C9—C5—C6	118.3 (4)	C14—C15—H15A	119.7
C9—C5—C4	123.1 (4)	C10—C15—H15A	119.7
C6—C5—C4	118.6 (4)		
			
C4—N1—N2—C3	173.3 (4)	C8—N4—C7—C6	0.8 (8)
C4—N1—N2—C1	1.8 (7)	C5—C6—C7—N4	−0.8 (9)
C3—N2—C1—C2	22.4 (6)	C7—N4—C8—C9	−0.1 (8)
N1—N2—C1—C2	−166.1 (4)	N4—C8—C9—C5	−0.4 (8)
N2—C1—C2—C10	−148.0 (4)	C6—C5—C9—C8	0.3 (7)
N2—C1—C2—S1	−27.1 (5)	C4—C5—C9—C8	−179.9 (5)
C3—S1—C2—C1	21.6 (3)	C1—C2—C10—C11	27.7 (7)
C3—S1—C2—C10	146.3 (4)	S1—C2—C10—C11	−90.6 (6)
N1—N2—C3—N3	0.9 (7)	C1—C2—C10—C15	−152.3 (5)
C1—N2—C3—N3	173.2 (5)	S1—C2—C10—C15	89.3 (5)
N1—N2—C3—S1	−177.7 (3)	C15—C10—C11—C12	5.0 (9)
C1—N2—C3—S1	−5.5 (5)	C2—C10—C11—C12	−175.1 (6)
C2—S1—C3—N3	171.0 (4)	C10—C11—C12—C13	−3.8 (11)
C2—S1—C3—N2	−10.3 (4)	C11—C12—C13—C14	0.6 (11)
N2—N1—C4—C5	178.3 (4)	C12—C13—C14—C15	1.4 (10)
N1—C4—C5—C9	−3.0 (7)	C13—C14—C15—C10	−0.1 (9)
N1—C4—C5—C6	176.7 (5)	C11—C10—C15—C14	−3.1 (9)
C9—C5—C6—C7	0.2 (8)	C2—C10—C15—C14	177.0 (5)
C4—C5—C6—C7	−179.6 (5)		

### Hydrogen-bond geometry (Å, º)

Cg2 and Cg3 are the centroids of the N4/C5–C9 pyridine and the C10–C15 phenyl ring, respectively.

**Table:**

*D*—H···*A*	*D*—H	H···*A*	*D*···*A*	*D*—H···*A*
O1—H1*C*···Br1	0.95	2.39	3.333 (4)	169
O1—H1*D*···Br1^i^	0.95	2.56	3.427 (5)	152
N3—H3*A*···Br1^ii^	0.90	2.45	3.333 (4)	167
N3—H3*B*···N4^iii^	0.90	1.99	2.840 (6)	158
C9—H9*A*···*Cg*2^iii^	0.93	2.96	3.650 (5)	132
C12—H12*A*···*Cg*3^iv^	0.93	2.82	3.565 (8)	137
C15—H15*A*···*Cg*3^v^	0.93	2.80	3.548 (6)	135

Symmetry codes: (i) *x*+1, *y*, *z*; (ii) *x*, *y*−1, *z*; (iii) −*x*+2, *y*−1/2, −*z*+2; (iv) −*x*+2, *y*−1/2, −*z*+1; (v) −*x*+1, *y*+1/2, −*z*+1.
